# Optimized solubility and bioavailability of genistein based on cocrystal engineering

**DOI:** 10.1007/s13659-023-00397-w

**Published:** 2023-09-13

**Authors:** Zhipeng Wang, Qi Li, Qi An, Lixiang Gong, Shiying Yang, Baoxi Zhang, Bin Su, Dezhi Yang, Li Zhang, Yang Lu, Guanhua Du

**Affiliations:** 1 Beijing City Key Laboratory of Polymorphic Drugs, Center of Pharmaceutical Polymorphs, Institute of Materia Medica, Chinese Academy of Medical Sciences and Peking Union Medical College, Beijing, 100050 People’s Republic of China; 2 Beijing City Key Laboratory of Drug Target and Screening Research, National Center for Pharmaceutical Screening, Institute of Materia Medica, Chinese Academy of Medical Sciences and Peking Union Medical College, Beijing, 100050 People’s Republic of China; 3 Shandong Soteria Pharmaceutical Co., Ltd., Laiwu, 271100 China

**Keywords:** Genistein, Piperazine, Cocrystal, Solubility, Bioavailability

## Abstract

With various potential health-promoting bioactivities, genistein has great prospects in treatment of a series of complex diseases and metabolic syndromes such as cancer, diabetes, cardiovascular diseases, menopausal symptoms and so on. However, poor solubility and unsatisfactory bioavailability seriously limits its clinical application and market development. To optimize the solubility and bioavailability of genistein, the cocrystal of genistein and piperazine was prepared by grinding assisted with solvent based on the concept of cocrystal engineering. Using a series of analytical techniques including single-crystal X-ray diffraction, powder X-ray diffraction, Fourier transform infrared spectroscopy, differential scanning calorimetry and thermogravimetric analysis, the cocrystal was characterized and confirmed. Then, structure analysis on the basis of theoretical calculation and a series of evaluation on the stability, dissolution and bioavailability were carried out. The results indicated that the cocrystal of genistein and piperazine improved the solubility and bioavailability of genistein. Compared with the previous studies on the cocrystal of genistein, this is a systematic and comprehensive investigation from the aspects of preparation, characterization, structural analysis, stability, solubility and bioavailability evaluation. As a simple, efficient and green approach, cocrystal engineering can pave a new path to optimize the pharmaceutical properties of natural products for successful drug formulation and delivery.

**Graphical Abstract:**

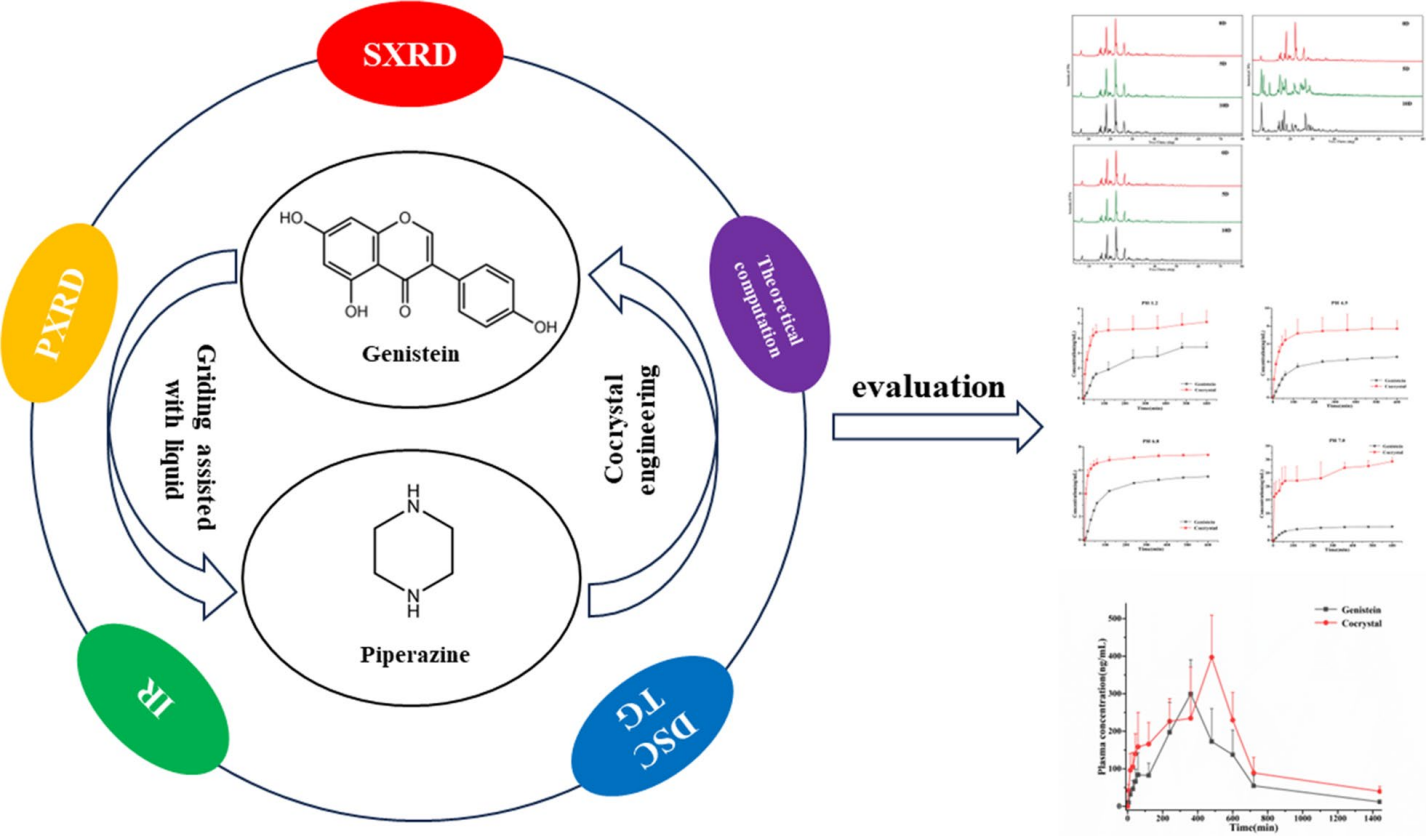

## Introduction

**Fig. 1:**
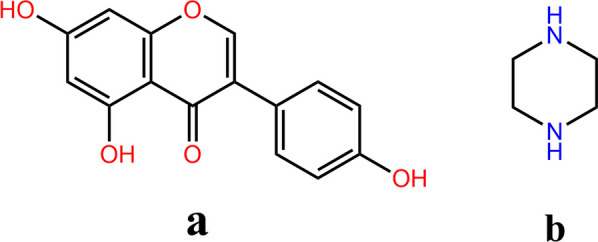
Chemical structures of genistein (**a**) and piperazine (**b**)

Genistein (GEN, Fig. 1a), a polyphenolic isoflavone with a rich plant-based source [1], is a significant member of natural products. Based on modern pharmacological studies, GEN is demonstrated to have a wide range of potential health-promoting bioactivities, including anti-oxidant [2], anti-inflammatory [3], anti-cancer [4, 5], anti-diabetic [6], hepatoprotective [7] and cardioprotective effects [8, 9]. Besides, GEN can lower blood lipids and blood pressure as well as treat osteoporosis and menopausal syndrome in women [10–12]. Unfortunately, GEN classified to Biopharmaceutical Classification System (BCS) Class II has the low aqueous solubility and poor bioavailability [13], which has great negative sides on its market development and clinical application.

In order to optimize the solubility and bioavailability of GEN, various approaches are adopted including salt formation [14], liposome [15, 16], nanosuspensions [17, 18] and other novel drug delivery systems such as solid lipid particulate system, super paramagnetic drug delivery system, hydrocolloids, polymeric micelles and so on [19]. Compared with these approaches, cocrystallization is a green, simple and promising route to improve the physiochemical and biopharmaceutical properties of drugs [20]. Without altering the pharmacological activity of drugs, cocrystal technology can optimize the physicochemical properties of drugs, such as mechanical properties, hygroscopicity, stability, solubility, permeability and bioavailability [21–23]. It is a significant step for pharmaceutical cocrystal development to select appropriate pharmaceutically acceptable cocrystal formers (CCFs) based on scientific and rational cocrystal design.

Due to the superior solubility, high safety and strong hydrogen bonding sites, piperazine (PPZ, Fig. 1b) is very popular to be regarded as CCF in the cocrystal design of weak acid drugs. As an important class of nitrogen heterocycles, the introduction of piperazine ring can effectively regulate the lipid-water partition coefficient and acid–base equilibrium constants of drugs, which is of great value in improving the solubility and pharmacokinetic properties of drugs. Nowadays, there are a variety of cocrystals with PPZ to be reported to achieve significant gains in solubility and bioavailability when compared with active pharmaceutical ingredients (APIs) prior to forming cocrystals [24–27]. Therefore, PPZ was selected as CCF to prepare cocrystal with GEN to improve solubility and bio-absorption of GEN in this study.

Over the past decades, some studies on the cocrystal of GEN were reported [28–34]. Among these reports, the majority of them were focused on the preparation, characterization and structural analysis of the GEN cocrystals. There are almost no studies to systematically and comprehensively launch an investigation from the aspects of preparation, characterization, structural analysis, stability, solubility and bioavailability evaluation. In this study, the cocrystal between GEN and PPZ was prepared by grinding assisted with solvent. Combining single-crystal X-ray diffraction analysis (SXRD), powder X-ray diffraction analysis (PXRD), infrared spectra analysis (IR), differential scanning calorimetry analysis (DSC), thermogravimetric analysis (TG) and theoretical computation, the prepared cocrystal were further characterized and validated. On the basis of confirming the formation of cocrystal and structural characterization, a series of evaluations in vitro and in vivo including stability, dissolution and bioavailability were launched systematically. The results indicated that the application of cocrystal technology achieved improved solubility and dissolution as well as optimized bioavailability of GEN. Targeting at the pharmaceutical defects of GEN, the enhancement of the solubility and bioavailability of GEN is realized by a clever and rational cocrystallization strategy, which not only devotes assistance to the market development and clinical application of GEN, but also provides a new path and option for the optimization of the activity of natural products.

## Results and discussion

### SXRD analysis

**Fig. 2:**
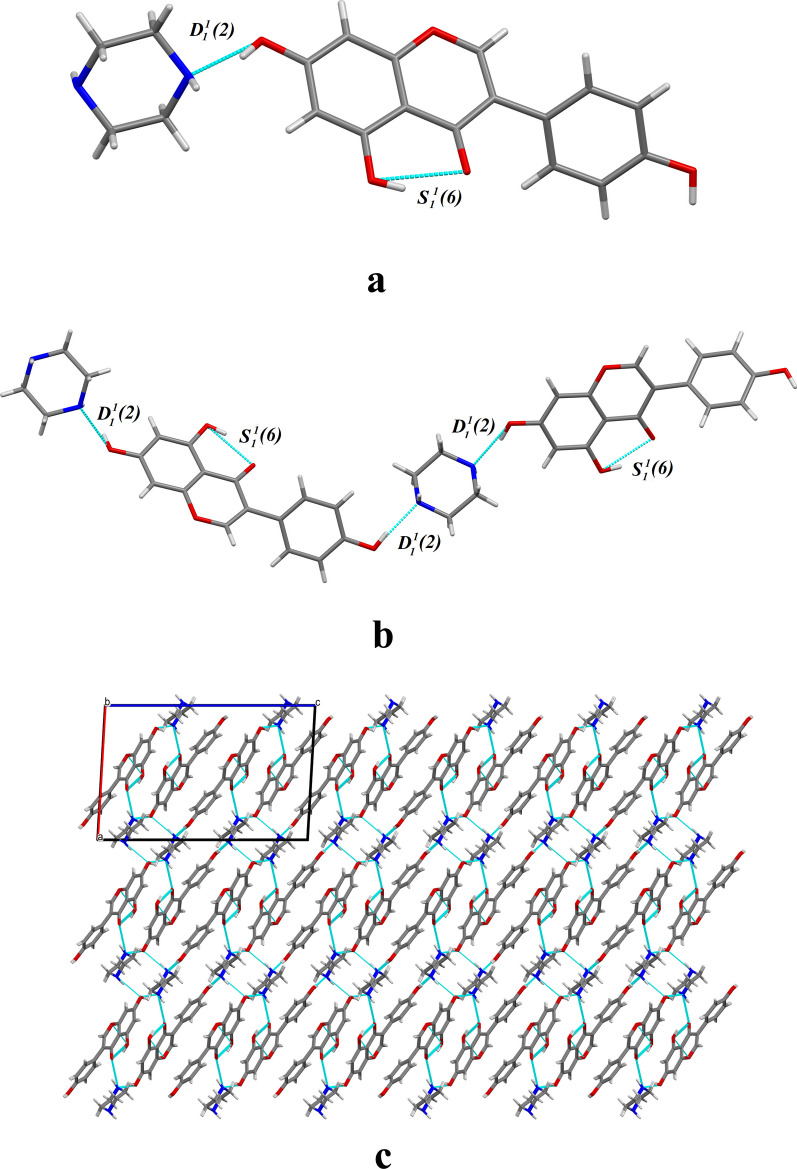
Crystal structure of the cocrystal of genistein and piperazine: **a** asymmetric unit, **b** a 1D hydrogen bonded chain, **c** 2D layer viewed into the b axis

As the gold standard for determining the three-dimensional structure of a molecule, SXRD analysis can provide various important information to characterize and confirm the molecular structure of cocrystals, which is also regarded as the most authoritative characterization tool for the identification of cocrystals. As listed in Table 1, the crystal structure of GEN-PPZ belongs to the monoclinic crystal system, P21/C space group. In the asymmetric unit, there are one GEN and one PPZ (Fig. 2a). Each GEN interacts with two PPZ and each PPZ also interacts with two GEN through O–H···N hydrogen bonds (Fig. 2b). With the help of two hydrogen bonds including O4–H4···N1P (2.576 Å) and O5–H5···N2P (2.729 Å), GEN and PPZ are linked in head-to-tail sequence to form a chain structure. Thanks to the effort of N1P–H1P···O2 (3.071 Å) and N2P–H2P···O4 (3.139 Å) hydrogen bonds, the formed chain structure connects to each other to generate a neat laminar structure from the view of b axis (Fig. 2c). Crystallographic data and significant refinement parameters of GEN-PPZ cocrystal structure were listed in Table 1 and the relative information on hydrogen bonds were given in Table 2. To our best known, the structure of drugs determines their properties. Along with the generation of GEN-PPZ cocrystal, the participation of various hydrogen bonds and the introduction of hydrophilic piperazine rings might contribute to the improvement of solubility of GEN, which was further proved by the powder dissolution study of GEN and its cocrystal.

**Table 1:** Crystal data and structure refinement parameters of genistein-piperazine cocrystal

Parameters	Genistein-piperazine cocrystal
Empirical formula	C_19_H_20_N_2_O_5_
Formula weight	356.37
Crystal size/mm	0.10 × 0.12 × 0.25
Description	Block
Crystal system	Monoclinic
Space group	P2_1_/_C_
a (Å)	12.307(1)
b (Å)	6.426(1)
c (Å)	21.394(1)
β (◦)	93.620(2)
Volume (Å3)	1688.7(1)
Z	4
Completeness	99.9%
Density (g cm^−3^)	1.402
Reflections with I > 2σ (I)	2975
Rindexs (I > 2σI)	R1 = 0.0380, wR2 = 0.1061
Goodness-of-fit on F2	1.055
CCDC deposition number	2286802

**Table 2:** Hydrogen bonds of genistein-piperazine cocrystal

D–H···A	d(D···A) (Å)	∠(DHA) (deg)	Symmetry code
O3–H3···O2	2.606	147.79	–
O4–H4···N1P	2.576	161.92	–
O5–H5···N2P	2.729	172.30	[x − 1, − y + 1/2, z + 1/2]
N1P–H1P···O2	3.071	143.03	[− x + 1, y − 1/2, − z + 1/2]
N2P–H2P···O4	3.139	167.06	[− x + 2, y − 1/2, − z + 1/2]

### PXRD analysis

**Fig. 3:**
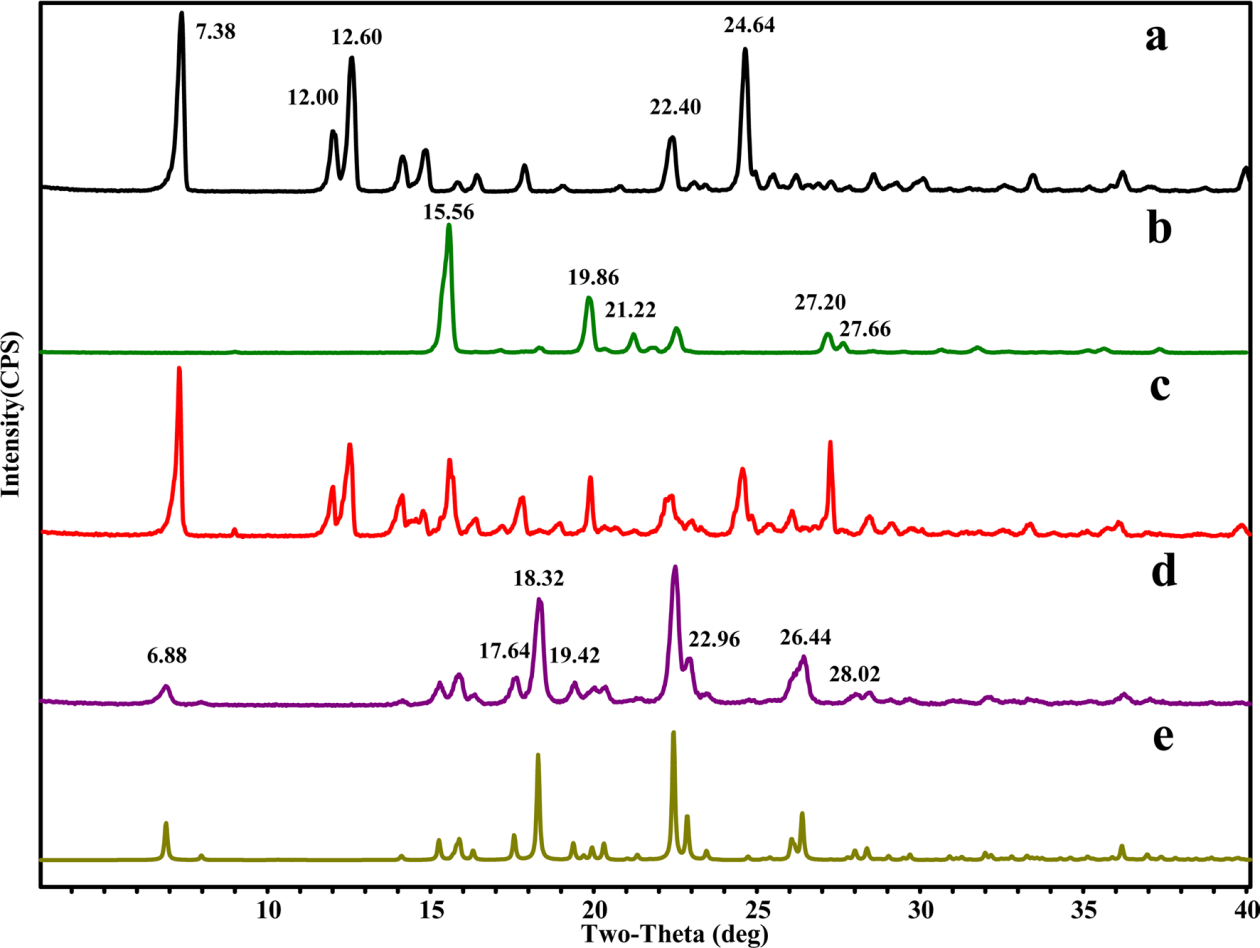
X-ray powder diffraction patterns of genistein (**a**), piperazine (**b**), physical mixture of genistein and piperazine (**c**), the cocrystal of genistein and piperazine prepared from grinding (**d**), the cocrystal of genistein and piperazine calculated from the single-crystal structure (**e**)

By comparing characteristic powder patterns, PXRD analysis is regarded as a powerful and fundamental tool to identify the solid states of compounds or complexes. As depicted in Fig. 3, GEN showed characteristic crystalline peaks at 2θ values of 7.38°, 12.00°, 12.60°, 22.42° and 24.64°, whereas PPZ showed characteristic crystalline peaks at 15.56°, 19.86°, 21.22°, 27.20° and 27.66°. However, GEN-PPZ cocrystal exhibited a unique PXRD pattern which was distinct from those of GEN and PPZ. In the PXRD pattern of GEN-PPZ cocrystal, new peaks emerged at 2θ values of 6.88°, 16.34°, 17.64°, 18.32°, 19.42°, 22.96°, 26.44°, 28.02° and 28.48°. The absence of characteristic peaks of both GEN and PPZ as well as the appearance of new peaks indicated that a new crystalline phase had generated. Besides, the simulated powder patterns calculated from the SXRD data matched well with the experimental pattern, which further confirmed the generation of GEN-PPZ cocrystal.

### IR analysis

**Fig. 4:**
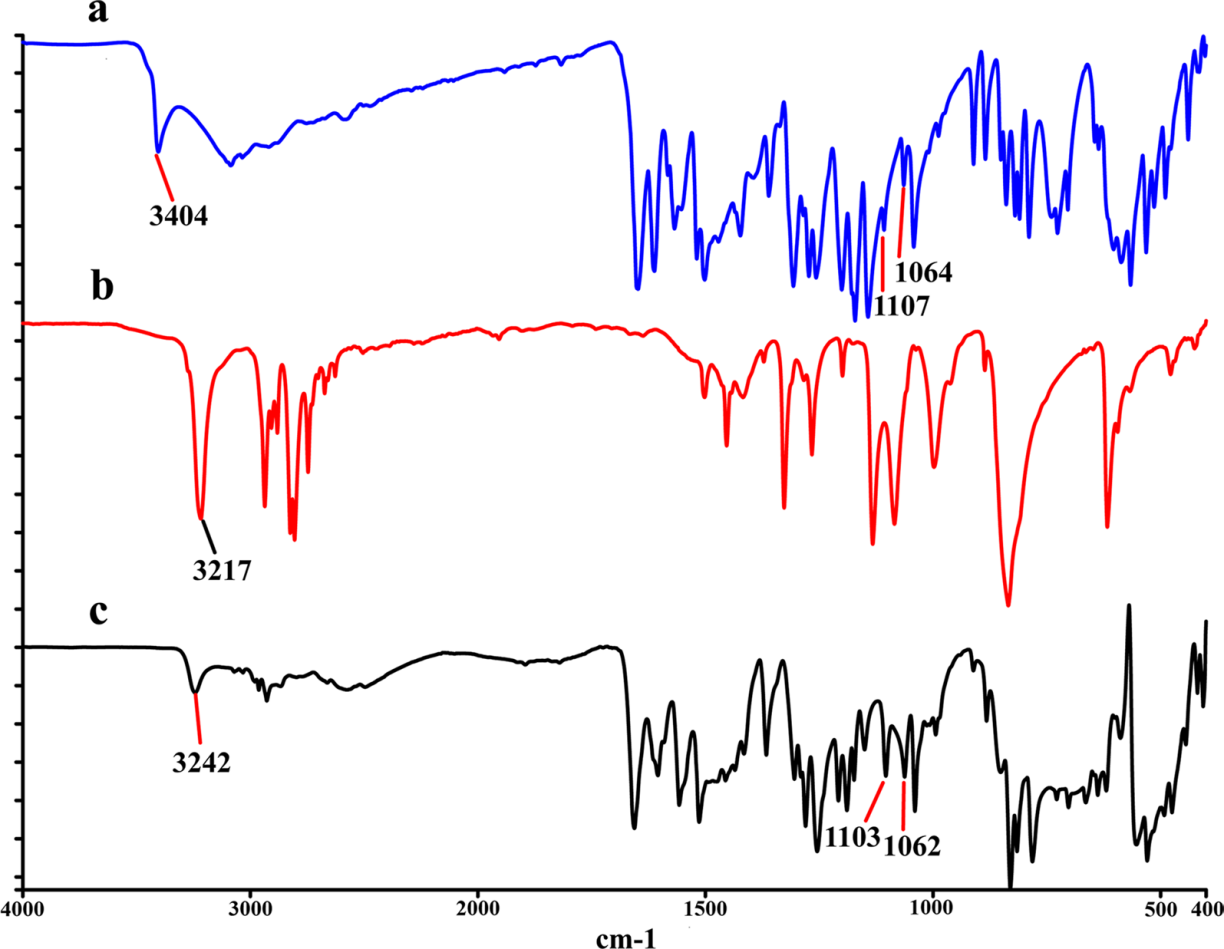
IR spectra of genistein (**a**), piperazine (**b**) and the cocrystal of genistein and piperazine (**c**)

To investigate the changes on noncovalent interactions within the formation of cocrystal, the IR study on the GEN-PPZ cocrystal was performed. The IR spectrum of GEN (Fig. 4a) depicted that the free O–H stretching absorption peak was observed at 3404 cm^−1^ and the C–O stretching absorption peaks were observed at 1107 and 1064 cm^−1^. Besides, the IR spectrum of PPZ (Fig. 4b) exhibited the free N − H stretching absorption peak was observed at 3217 cm^−1^. In the IR spectrum of GEN-PPZ cocrystal (Fig. 4c), absorption peaks of GEN (3404, 1107 and 1064 cm^−1^) and PPZ (3217 cm^−1^) disappeared and some new absorption peaks at 3242, 1103 and 1062 cm^−1^ emerged, which was attributed to the intermolecular hydrogen bonding (O–H···N) between GEN and PPZ.

### Thermal analysis

**Fig. 5:**
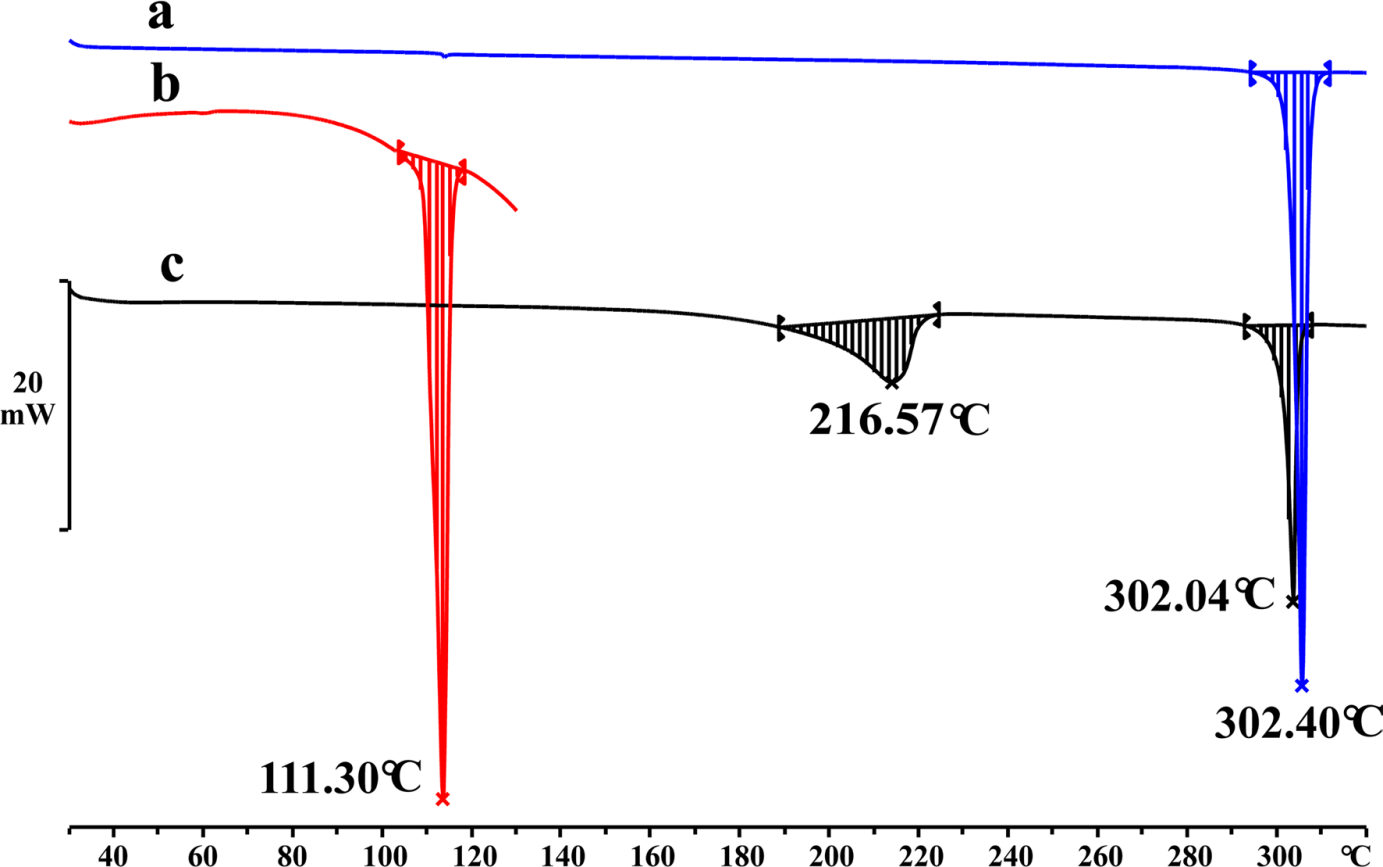
DSC results of genistein (**a**), piperazine (**b**) and the cocrystal of genistein and piperazine (**c**)

**Fig. 6:**
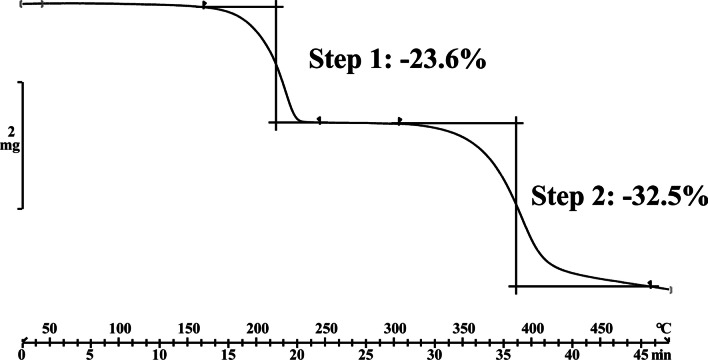
TG results of the cocrystal of genistein and piperazine

As a sensitive technology to analyze the transformation of material phase, DSC also plays significant roles in the characterization of cocrystal. As was presented in Fig. 5, the sharp endothermic peak of GEN-PPZ cocrystal at 216.57 °C was distinct from the peaks of GEN (302.40 °C) and PPZ (111.30 °C), which collaboratively confirmed the generation of the new solid phase. However, there was an interesting phenomenon that the cocrystal had two endothermic peaks at 216.57 °C and 302.04 °C. The sharp endothermic peak at 302.04 °C was quite close to the melting point of GEN (302.40 °C). It was predicted that the cocrystal decomposed at 216.57 °C. The cocrystal was separated into GEN and PPZ. With the increase of temperature, there was only GEN and PPZ was sublimated due to its low boiling point at about 146.0 °C. Combined with the result of TG, this hypothesis was further confirmed. In Fig. 6, there were two distinct weight loss steps with the increasing heating. The first weight loss of 23.6% from 162 to 224 °C corresponded to the loss of PPZ molecule (theoretical weight percentage is 24.1%). Besides, the phenomenon was consistent with the previous study [34]. According to the result of TG analysis, there was no mass loss to be found in the test temperature range below the melting point of GEN-PPZ cocrystal, which indicated that no solvent molecules were present in the lattice of the cocrystal.

### Theoretical computation

**Fig. 7:**
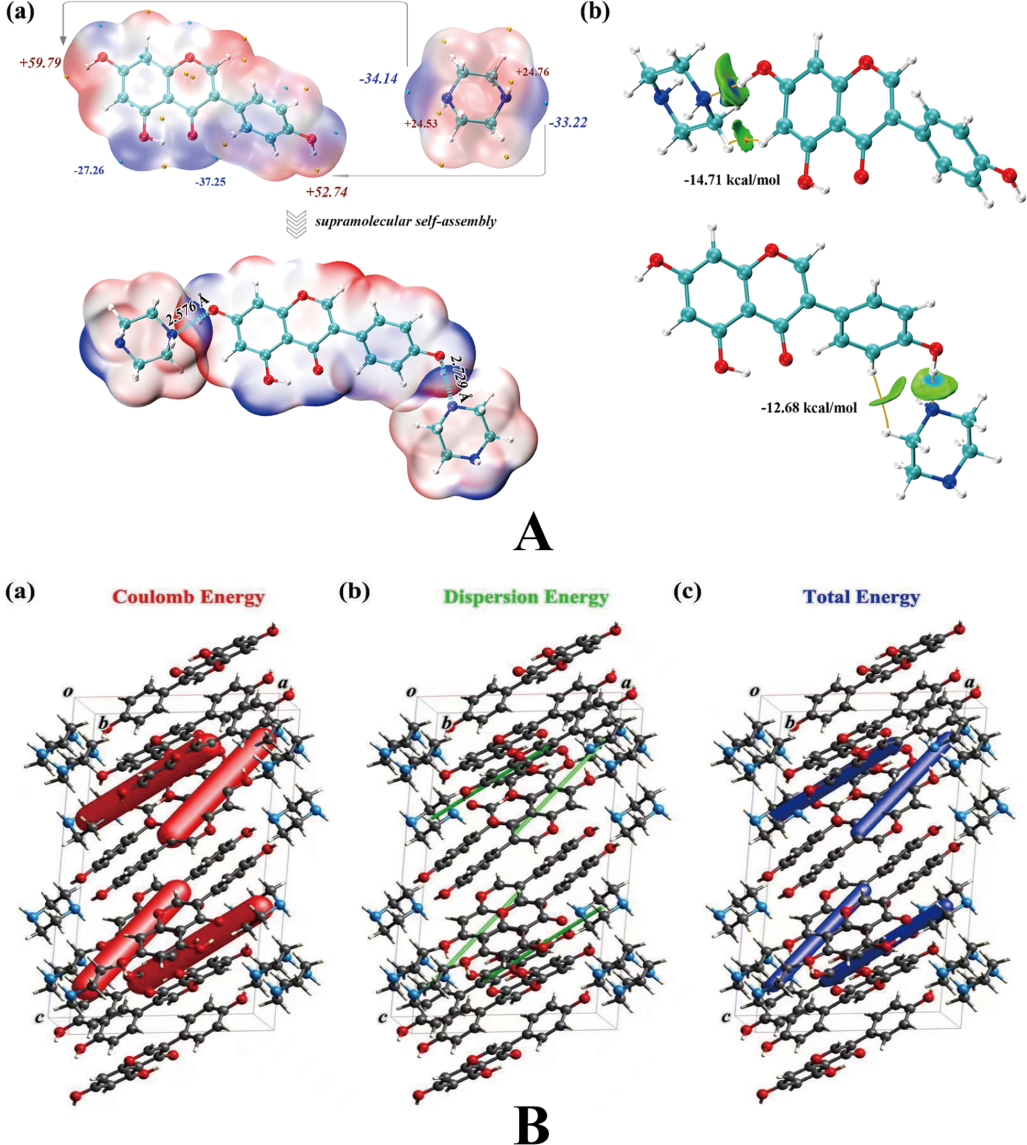
The results of MEPS analysis (**A**-**a**), interaction energy analysis (**A**-**b**) and energy framework analysis (**B**)

As a favorable tool, MEPS analysis was adopted to analyze the capabilities of molecules as hydrogen bond donors and acceptors. As presented in Fig. 7A(a), the first global maxima site of GEN appeared on the site of O4 (59.79 kcal mol^−1^) and the first global minima sites of PPZ appeared on the site of N1P (− 34.14 kcal mol^−1^). Likewise, the secondary global maxima site of GEN appeared on the site of O5 (52.74 kcal mol^−1^) and the secondary global minima sites of PPZ appeared on the site of N2P (− 33.22 kcal mol^−1^). Based on the hierarchical organization of the functional-group interaction theory, the main interaction sites in the cocrystal should first occur pairwise in the minima and maxima of the MEPS, followed by the secondary ones. Hence, the interaction of O4–H4···N1P was strongest and the interaction of O5–H5···N2P was inferior, which was corroborated by their individual bond length (O4–H4···N1P was 2.576 Å and O5–H5···N2P was 2.729 Å). The result of interaction energy analysis (Fig. 7A(b)) further demonstrated that the strength of molecular interactions between O4 and N1P was strongest and the strength of molecular interactions between O5 and N2P was the secondary. These analysis results were consistent with the fact that O4–H4···N1P and O5–H5···N2P mainly keep the basic structure of cocrystal. Besides, the result of energy framework analysis (Fig. 7B) indicated that coulomb energy occupied the majority of the total energy and dispersion energy only taken a small part of the total energy, which suggested that hydrogen bonding forces played a dominant role and van der Waals forces played a secondary role in keeping spatial arrangement of cocrystal molecule.

### Stability study

**Fig. 8:**
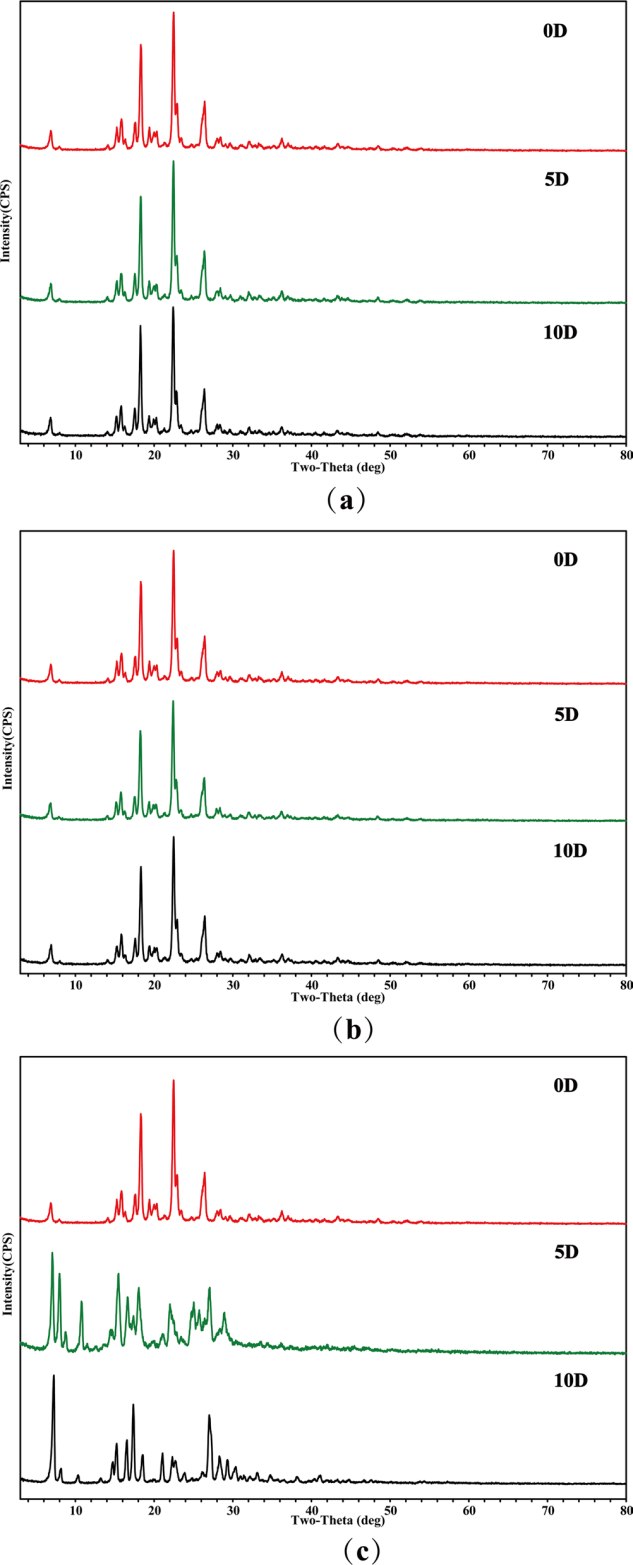
PXRD comparisons on the stability study of the cocrystal between genistein and piperazine under high-temperature (**a**), illuminated environments (**b**) and high-humidity (**c**)

Stability is closely related to the quality and safety of drugs, involving multiple links such as drug production, storage, transportation and so on, which is of great significance for drug quality evaluation. In this study, the stability of GEN-PPZ cocrystal was evaluated in high-temperature, high-humidity and illuminated environments on the days 0, 5 and 10, respectively. The results indicated that there was almost no change to be observed in the PXRD pattern of the cocrystal under high-temperature and illuminated environments within 10 days (Fig. 8), which demonstrated that the cocrystal could maintain stability under high-temperature and illuminated environments. Unsatisfactorily, there was significant changes in the PXRD pattern of the cocrystal under high humidity within 5 and 10 days, which suggested the cocrystal could not keep stable in high humidity. In order to overcome this shortcoming, adding desiccants, coating and other preparation processes could be taken into consideration in the development of the cocrystal.

### Powder dissolution in vitro

**Fig. 9:**
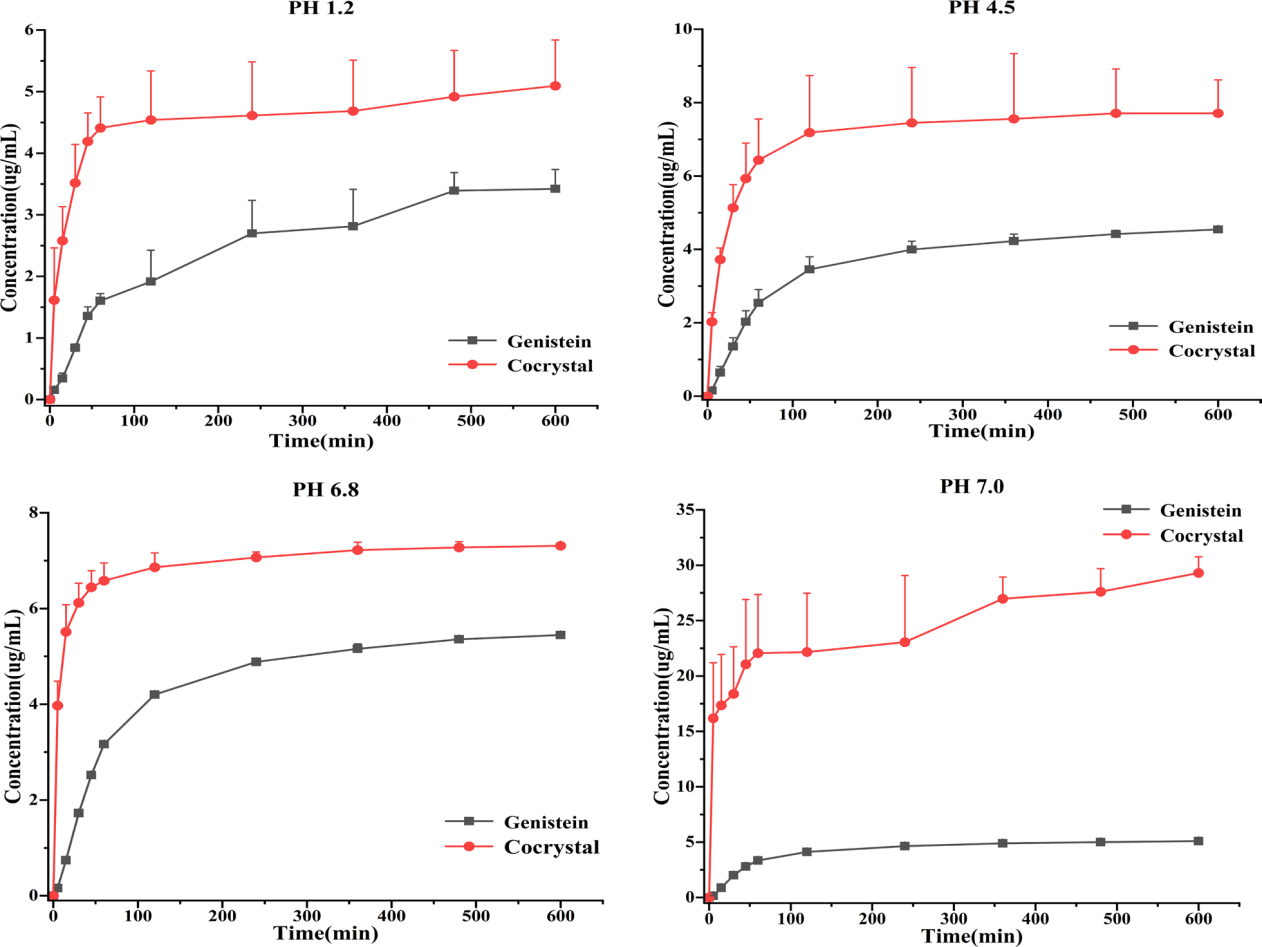
Powder dissolution of the pure genistein and the cocrystal of genistein and piperazine in four different media (pH = 1.2, pH = 4.5, pH = 6.8 and pH = 7.0)

Poor water solubility, the most important drug-forming defect of GEN, is a major problem that needs to be solved. Employing cocrystal technology, an investigation with the purpose of optimizing the solubility of GEN was carried out in this study. In the case of pure GEN, the maximum concentration was individually 3.42, 4.55, 5.45 and 5.10 μg/mL in four different mediums (pH 1.2, pH 4.5, pH 6.8 and pH 7.0) after 10 h. Correspondingly, the maximum concentration of GEN in the case of cocrystal was respectively 5.09, 7.71, 7.31 and 29.31 μg/mL in four different mediums after 10 h. Compared with the parent drug, the concentration of GEN released from the GEN-PPZ cocrystal after 10 h was respectively 1.5, 1.7, 1.4 and 5.7-fold that of the GEN in four different mediums. The powder dissolution profiles (Fig. 9) also illustrated that the GEN-PPZ cocrystal possessed a faster dissolution rate than pure GEN. Especially, the GEN-PPZ cocrystal achieved surprising elevation in the aspect of solubility and dissolution rate in distilled water (pH = 7.0), which might effectively increase the absorption and bioavailability of GEN. The ability of cocrystal to exhibit superior solubility compared with pure GEN might be related to the following three reasons. Firstly, the generation of cocrystal introduced the participation of a variety of hydrogen bonds that increased the opportunity to interact with water molecules. Secondly, the introduction of the hydrophilic piperazine ring resulted in enhanced hydrophilicity of the entire cocrystal molecule. Thirdly, the alkaline microenvironment created by PPZ improved the solubility of GEN which was attributed to the physical property of higher solubility of GEN in dilute alkaline conditions compared to neutral and acidic conditions.

### Pharmacokinetics in vivo

**Fig. 10:**
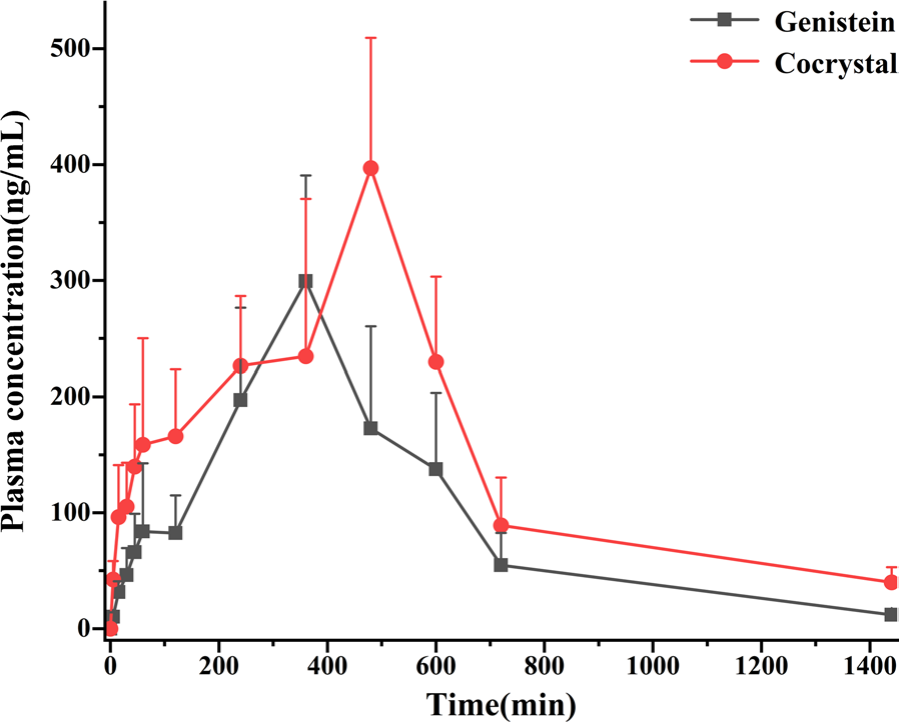
Plasma concentration–time profiles of genistein and genistein from the cocrystal after oral administration

The plasma concentration–time profiles of GEN-PPZ cocrystal and pure GEN after oral administration were depicted in Fig. 10. The calculated pharmacokinetic parameters were summarized in Table 3. Compared with the Cmax of pure GEN (307.49 ± 93.33 μg/mL), the Cmax of cocrystal reached 413.71 ± 105.32 μg/mL, which achieved a large boost. By the measurement of AUC_0-∞_, the bioavailability of GEN from cocrystal was 161% of that of GEN alone, which suggested that the formation of cocrystal had positive effects on improving the bioavailability of GEN. Additionally, delayed T_max_ and t_1/2_ of GNE from the cocrystal indicated that the formation of cocrystal prolonged action time of GEN in vivo. At the same time, increased MRT_0-∞_ and decreased CLz/F gave us information that GEN from the cocrystal owned longer retention time and slower rate of elimination in vivo when compared to pure GEN. It was reported that GEN had severe first-pass elimination and high safety [35–38]. In this study, the generation of cocrystal not only reduced the elimination of GEN, but also increased its duration of action in vivo, which was beneficial for the played efficacy of GEN in vivo. Based on the above analysis, it could be predicted that the increased bioavailability of GEN with the help of cocrystal technology might have close association with elevated solubility, increased C_max_, prolonged duration of action and attenuated elimination.

**Table 3:** Pharmacokinetic parameters after oral administration of genistein and genistein-piperazine cocrystal in rats (x ± s, n = 6)

Parameter	Genistein	Genistein-piperazine
AUC(0-t) (ug/L*h)	2280.34 ± 296.29	3475.29 ± 598.98**
AUC(0-∞) (ug/L*h)	2355.71 ± 273.25	3791.34 ± 814.75**
MRT(0-t) (h)	7.63 ± 0.71	8.50 ± 0.26*
MRT(0-∞) (h)	8.41 ± 1.16	10.61 ± 0.91**
t1/2z (h)	4.02 ± 1.71	5.58 ± 1.94
T_max_ (h)	5.67 ± 0.82	7.33 ± 1.03*
CLz/F (L/h/kg)	42.95 ± 5.16	36.28 ± 8.44
Vz/F (L/kg)	254.39 ± 117.43	274.61 ± 48.07
C_max_ (ug/L)	307.49 ± 93.33	413.71 ± 105.32

**P < 0.01 between GEN and GEN-PPZ, *P < 0.05 between GEN and GEN-PPZ

## Experimental section

### Compounds and agents

GEN (purity > 98%, MW = 270.24) was purchased from Shanxi Huike Plant Development Co., Ltd. (Shanxi, China), daidzein (purity > 98%, MW = 254.24) was purchased Wuhan Yuancheng Technology Development Co., Ltd. (Wuhan, China) and PPZ (purity > 99%, MW = 86.14) was purchased from Nine-Dinn Chemistry Co., Ltd. (Shanghai, China). All solvents used for crystallization were of analytical grade and purchased from the Sinopharm Chemical Reagent Beijing Co., Ltd.

### Preparation of GEN-PPZ cocrystal

To prepare GEN-PPZ cocrystal, 0.2 mmol of GEN and 0.2 mmol of PPZ were accurately weighed into a mortar, 1 mL of acetone was added and the solid was manually grounded at an ambient temperature for 15 min. The solid was dried at an ambient temperature for 4 h to obtain an auratus powder sample.

### Preparation of single crystal

The crystal of GEN-PPZ cocrystal was prepared with reaction crystallization. 0.2 mmol of GEN (54.0 mg) and 0.2 mmol of PPZ (17.2 mg) were accurately weighed into a vial, 10 mL of ethanol was added and the mixture was stirred at an ambient temperature at 300 rpm for 48 h. The solution was filtered to obtain clear and transparent filtrates. The obtained filtrates were evaporated at 25 °C and colorless block crystals were obtained after 10 days.

### Single-crystal X-ray diffraction (SXRD)

The SXRD of GEN-PPZ cocrystal was carried out on a Rigaku XtaLAB Synergy four-circle diffractometer (Rigaku, Americas) with Cu–Kα radiation (wλ = 1.54178 Å) at the temperature of 293 K. The structure of qualified single crystal sample obtained in this study was solved by direct method and refined with the full-matrix least-squares technique. The non-hydrogen atoms were refined with anisotropic displacement parameters and hydrogen atoms were placed at the calculated positions and refined with a riding model. The related SXRD data was analyzed with the OLEX2 program and MERCURY software (Version 2023.1.0) was employed to prepare molecular diagrams.

### Powder X-ray diffraction (PXRD)

In this study, a Rigaku D/max-2550 diffractometer (Rigaku, Tokyo, Japan) equipped with a Cu–Kα radiation source set at 40 kV and 150 mA was employed to launch the PXRD experiments. With a scan rate of 8°/min, the diffraction data were collected in the 2θ range of 3–40°. All the data were analyzed and processed with Jade 6.0 software.

### Infrared spectra (IR)

Infrared spectra were recorded using a PerkinElmer FTIR spectrophotometer (PerkinElmer, USA) with an attenuated total reflectance sampling accessory. The wavelength number ranged from 4000 to 400 cm^−1^ with 16 scans at a resolution of 4 cm^−1^.

### Thermal analysis

To characterize the samples in this study, a Mettler Toledo DSC/DSC 1 (Mettler Toledo, Greifensee, Switzerland) was employed. The accurately weighed samples in hermetically sealed aluminum crucibles with a pinhole were heated from 30 °C to specified temperature at a constant rate of 10 °C/min under a nitrogen gas flow of 50 mL/min.

With a Mettler Toledo DSC/TGA1 (Mettler Toledo, Greifensee, Switzerland), the TGA analysis on the sample was launched. The accurately weighed sample in aluminum oxide crucible was heated from 30 to 500 °C at a constant ramp of 10 °C/min under a nitrogen gas flow of 50 mL/min.

The obtained DSC and TG data were analyzed and processed with the STAR software package (STARe Default DB V9.10, Mettler Toledo, Greifensee, Switzerland).

### Theoretical computation

The theoretical levels of geometry optimizations and single-point energy calculation were B3LYP-D3BJ/6-31G (d, p) and B3LYP-D3BJ/6-311 + G (2d, 2p) using Gaussian package [39]. Geometry optimizations were performed only for hydrogen atoms and the coordinates of heavy atoms were obtained from the experimental of SXRD [40]. With counterpoise corrections method, the interaction energies of GEN-PPZ cocrystal were calculated at the same level used in single-point energy calculation [41]. Besides, the intermolecular interactions existing in GEN-PPZ cocrystal were investigated with independent gradient model based on Hirshfeld partition (IGMH) method using Multiwfn program [42]. Molecular electrostatic potential surface (MEPS) analysis and energy framework analysis were launched by CrystalExplorer (version 21.5) [43].

### Stability study

To evaluate the stability of GEN-PPZ cocrystal with PXRD analysis, approximately 50 mg cocrystal samples in open containers were placed in the conditions of high temperature (60 ± 2 °C), high humidity (25 ± 2 °C, 90 ± 5%) and illumination (4500 ± 500 lx) for 10 days.

### Powder dissolution in vitro

Before the experiment of dissolution, the samples should be processed with a 100-mesh sieve in advance to minimize the influence of sample particle size on the results of experiments. Performed on an RC12AD (Tianjin TIANDA TIANFA pharmaceutical testing instrument manufacturer, Tianjin, China) with an automatic sampling system RZQ-12D (Tianjin TIANDA TIANFA pharmaceutical testing instrument manufacturer, Tianjin, China), the dissolution study of GEN and GEN-PPZ cocrystal was investigated. In this device with automatic fluid replenishment, an equal volume of the blank dissolution media can be injected automatically into the dissolution vessels after sampling. Accurately weighed GEN (60.0 mg) and GEN-PPZ cocrystal (79.2 mg, which is equivalent to 60.0 mg GEN) were added to dissolution vessels containing 900 mL media, including 0.1 M hydrochloric acid aqueous solution (pH = 1.2), acetate buffer (pH = 4.5), phosphate buffer (pH = 6.8) and pure water (pH = 7.0). Samples were stirred at 100 rpm at 37 °C and 1 mL of sample was obtained at 0, 5, 15, 30, 45, 60, 120, 240, 360, 480 and 600 min. Then, the concentration of GEN in these samples was determined by HPLC. The liquid-phase conditions were as follows: Welch Ultimate AQ-C18 (250 mm × 4.6 mm, 5 µm); mobile phase, methanol–0.1% acetic acid (70:30, v/v); detection wavelength, 260 nm; flow rate, 1.0 mL/min; column temperature, 30 °C; injection volume, 10 µL.

### Pharmacokinetic study in vivo

A total of 12 male pathogen-free Sprague–Dawley rats (230 ± 20 g) were kindly supplied by the Experimental Animal Center of the Institute of Materia Medica, Chinese Academy of Medical Sciences. Animals were housed and handled under suitable humidity, temperature and light. Rats were allowed to acclimate for a period of 1 week with free access to water and standard rodent food. All animal studies were carried out in accordance with the Guideline for Animal Experimentation of the Institute of Materia Medica, Chinese Academy of Medical Sciences and the protocol was approved by the Animal Ethics Committee of the institution.

Animals were randomly divided into two groups (n = 6 per group) and these two groups of SD rats were individually administrated with GEN (100 mg/kg) or GEN-PPZ cocrystal (131.9 mg/kg, which is equivalent to 100 mg/kg GEN). After administration, 400 μL blood samples were collected through retro-orbital venous plexus at 0, 5, 15, 30, 45, 60, 120, 240, 360, 480, 600, 720 and 1440 min. The samples were centrifuged at 4000 rpm (10 min, 4 °C) and the supernatants of samples were stored at − 80 °C until analysis.

After thawing plasma samples at room temperature, 100 μL plasma was mixed with 20 μL solution of daidzein (1.0 μg/mL, used as the internal standard, IS) in a 1.5 mL eppendorf tube. After vortex-mixing 1 min, 1.0 mL ethyl acetate and 20 μL 0.1 mol/L hydrochloric acid were added in order. Then, the mixture was vortexed for 5 min and centrifuged at 4000 rpm for 10 min. 800 μL supernatant was separated out and blown to dryness with nitrogen at 40 °C. 50 μL methanol was added and vortexed for 1 min. After centrifugation (10 min, 13,400 rpm), 10 μL of supernatant was injected into the HPLC for analysis. With the help of DAS 2.0 software, the plasma concentration–time profile and some significant pharmacokinetic parameters were obtained. In order to evaluate the differences in bioavailability between two groups, acquired pharmacokinetic parameters were processed with SPSS 21.0 (Statistical Package for the Social Science, Chicago, IL). The acquired data were expressed as the mean ± standard deviation (mean ± SD). Besides, the difference was considered statistically significant when *p* value < 0.05.

## Conclusion

On the basis of the design concept of cocrystal engineering, a novel cocrystal composed of GEN and PPZ at a 1:1 ratio was successfully prepared to optimize the solubility and bioavailability of GEN. Systematic characterizations combining various analytical technology including SXRD, PXRD, IR, DSC and TG consistently provide strong identifications and confirmation to the formation of the new solid form. With the help of SXRD analysis and theoretical calculation, detailed structural information on the cocrystal was clarified and validated. The results of stability evaluation demonstrated that the cocrystal was stable under the high temperature and light condition and unstable at high humidity. The investigation of powder dissolution in vitro indicated that the generation of cocrystal significantly increased the solubility and dissolution rate. Besides, pharmacokinetics study in vivo also illustrated that cocrystal technology could improve the bioavailability of GEN, which would contribute to enhancing its efficacy. This study indicated that the cocrystallization as an effective technique had huge potential to optimize the pharmaceutical properties of natural products for successful drug formulation and delivery.

## Data Availability

The data supporting the findings of this study are available upon reasonable request from the corresponding author.
